# Supplementary material for: The cerebellum engages multiple pre-motor pathways through a divergent-convergent architecture to shape whisker dynamics
Source: Front Neural Circuits. 2026 Jan 22;19:1706704. doi: 10.3389/fncir.2025.1706704 (PMC12872751; doi:10.3389/fncir.2025.1706704)
Supplement: Supplementary file 1 [file Image_1.pdf]

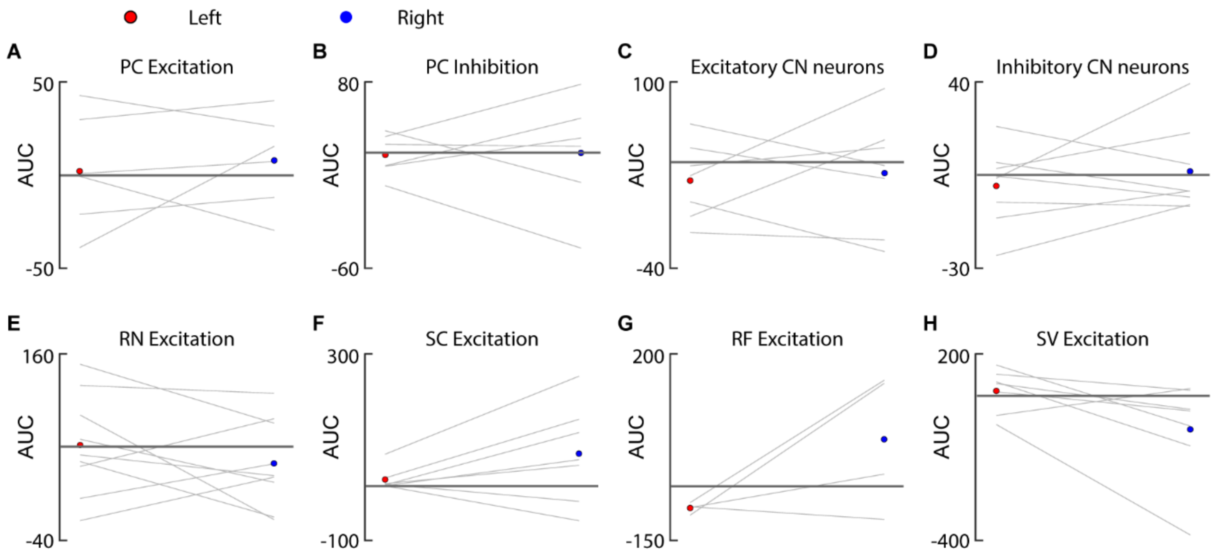

Figure S1

Figure S1: (A) Area under the curve (AUC) of the average velocity trace in the first 25 ms after stimulus onset during PC excitation. AUC is extracted for both the left and right whisker. Gray lines represent paired datapoints and the colored circles represent the average for left and right in red and blue, respectively. (**B**), (**C**), (**D**), (**E**), (**F**), (**G**), (**H**), are similar as (**A**), but for PC inhibition, stimulation of excitatory CN neurons, stimulation of inhibitory CN neurons, RN excitation, SC excitation, RF excitation, and SV excitation, respectively. Horizontal line represents value 0.

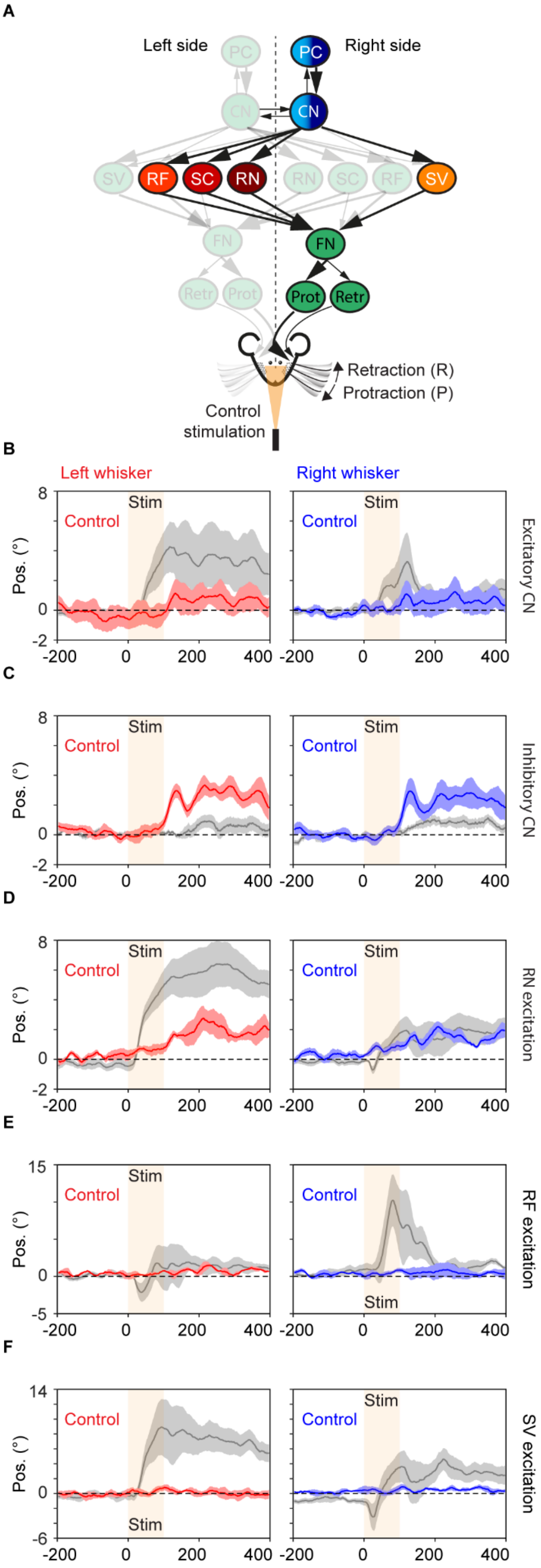
A
Left side
Right side
PC
CN
SV
RF
SC
RN
FN
Prot
Retr
Retraction (R)
Protraction (P)
Control
stimulation
B
Left whisker
Right whisker
Stim
Control
Pos. (°)
Excitatory CN
C
Inhibitory CN
D
RN excitation
E
RF excitation
F
SV excitation

Figure S2

Figure S2: (**A**) A schematic of the neural circuit with colours indicating the sites of stimulation. The orange light given as a control (visual) stimulus was placed in front of the mouse as indicated in the schematic. (**B**) Left: Average whisker movement of the left whisker aligned to either the real (stimulation of excitatory CN) or control stimulus in grey and red, respectively. The period of stimulation is highlighted by the orange shaded area. Right: Average whisker movement of the right whisker aligned to either the real (stimulation of excitatory CN) or control stimulus in grey and blue, respectively. The period of stimulation is highlighted by the orange shaded area. (**C**), (**D**), (**E**), and (**F**) are all similar to (**B**) but for the stimulation of inhibitory CN, RN excitation, RF excitation, and SV excitation, respectively. Abbreviation: Purkinje cell of the Paramedian lobule (PC), cerebellar nuclei (CN), facial nucleus (FN), reticular formation (RF), superior colliculus (SC), spinal trigeminal nucleus (SV), the red nucleus (RN), protractor muscles (Prot), retractor muscle (Retr), angle whisker position (Pos).

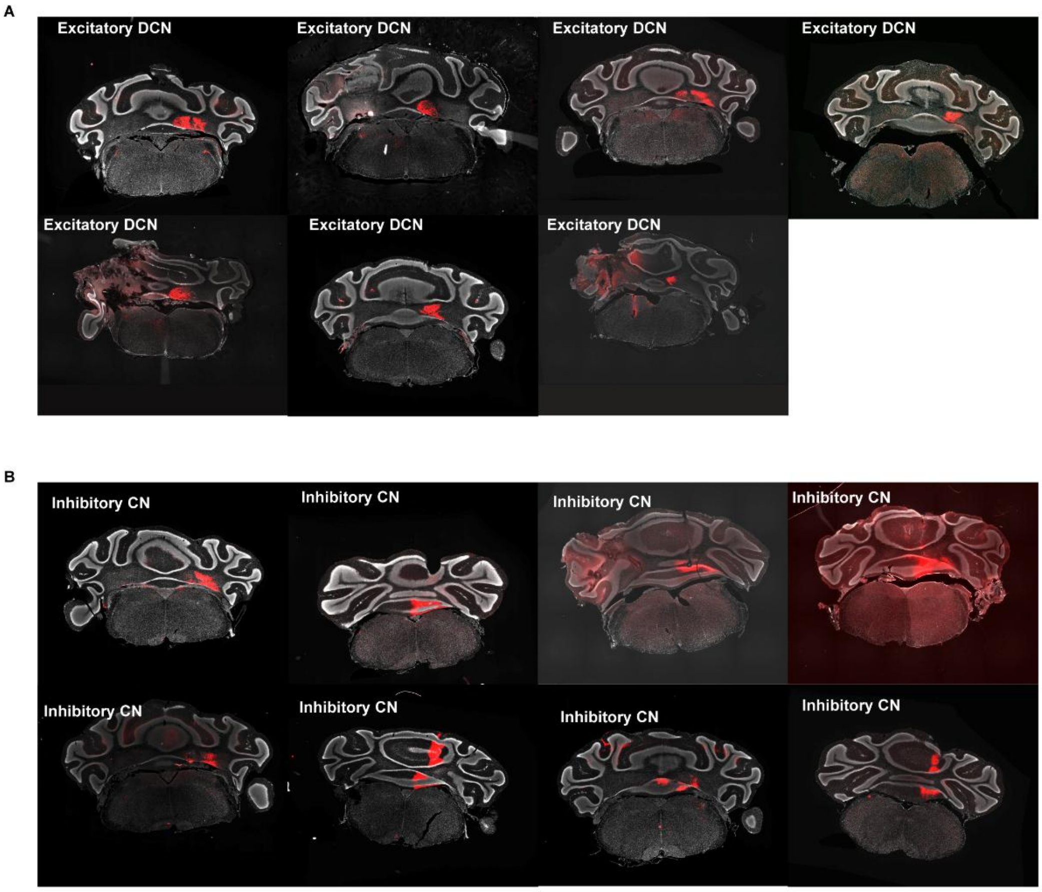

Figure S3

Figure S3: (A) Histological examination of transgene expression in excitatory (glutamatergic) CN of 7 mice. **(B)** Histological examination of transgene expression in inhibitory (GABAergic) CN of 8 mice.

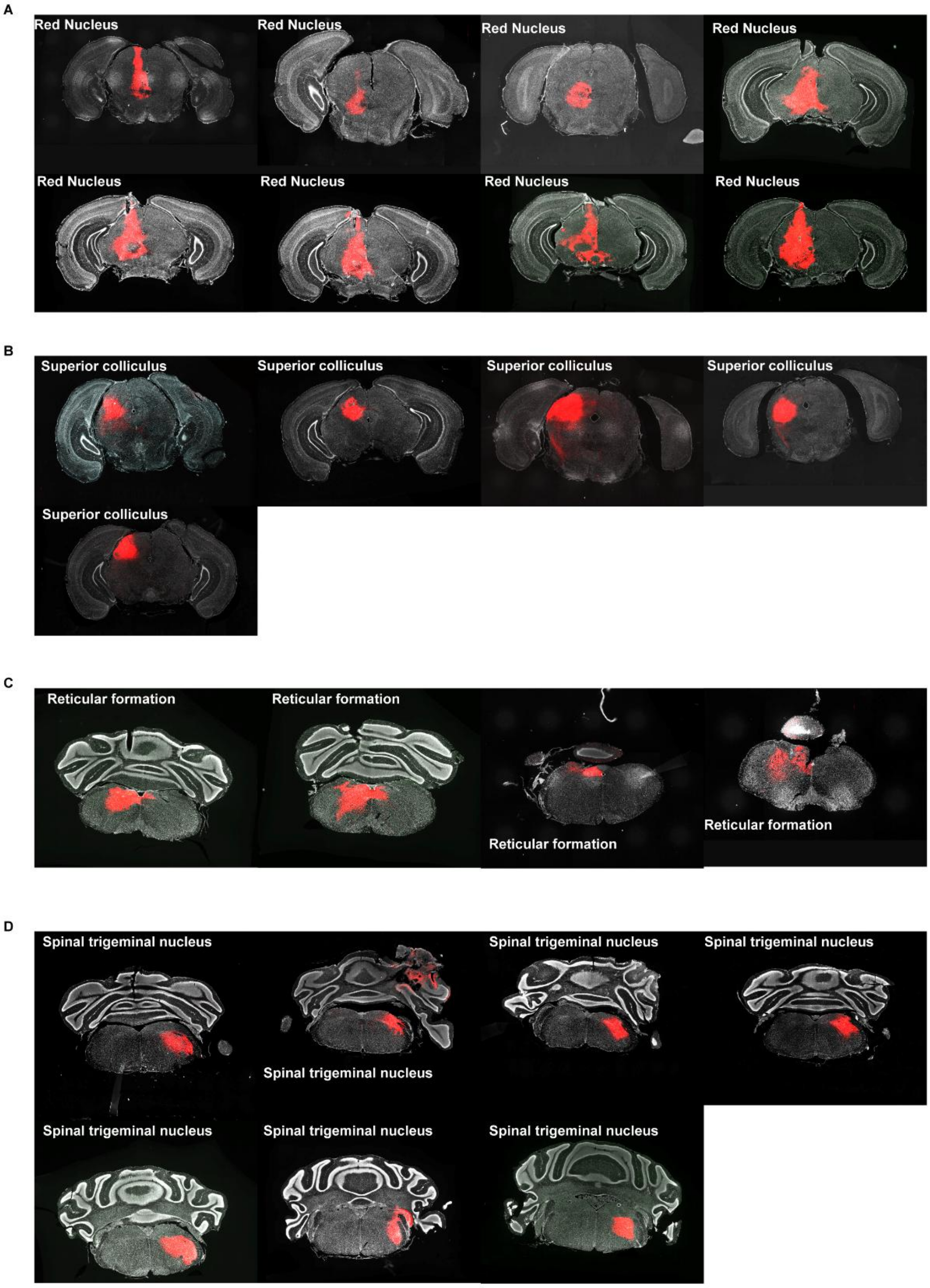

Figure S4

Figure S4: (A) Histological examination of transgene expression in the red nucleus of 8 mice. **(B)** Histological examination of transgene expression in the superior colliculus of 5 mice. **(C)** Histological examination of transgene expression in the reticular formation of 4 mice. **(D)** Histological examination of transgene expression in the spinal trigeminal nucleus of 7 mice.
